# Prediction of interactions between the SARS-CoV-2 ORF3a protein and small-molecule ligands using the ANDSystem cognitive platform, graph neural networks, and molecular modeling

**DOI:** 10.18699/vjgb-25-113

**Published:** 2025-12

**Authors:** T.V. Ivanisenko, P.S. Demenkov, M.A. Kleshchev, V.A. Ivanisenko

**Affiliations:** Institute of Cytology and Genetics of the Siberian Branch of the Russian Academy of Sciences, Novosibirsk, RussiaInstitute of Cytology and Genetics of the Siberian Branch of the Russian Academy of Sciences, Novosibirsk, RussiaInstitute of Cytology and Genetics of the Siberian Branch of the Russian Academy of Sciences, Novosibirsk, Russia; Institute of Cytology and Genetics of the Siberian Branch of the Russian Academy of Sciences, Novosibirsk, Russia; Institute of Cytology and Genetics of the Siberian Branch of the Russian Academy of Sciences, Novosibirsk, Russia; Institute of Cytology and Genetics of the Siberian Branch of the Russian Academy of Sciences, Novosibirsk, Russia

**Keywords:** ANDSystem, SARS-CoV-2, ORF3a, gene networks, graph neural networks, protein–ligand interaction prediction, bictegravirum, 4-(benzoylamino)benzoic acid, molecular docking, potential therapeutic agents, ANDSystem, SARS-CoV-2, ORF3a, генные сети, графовые нейронные сети, предсказание белок–лиганд взаимодействий, биктегравир, 4-(бензоиламино)бензойная кислота, молекулярный докинг, потенциальные лекарства

## Abstract

In recent years, artificial intelligence methods based on the analysis of heterogeneous graphs of biomedical networks have become widely used for predicting molecular interactions. In particular, graph neural networks (GNNs) effectively identify missing edges in gene networks – such as protein–protein interaction, gene–disease, drug–target, and other networks – thereby enabling the prediction of new biological relationships. To reconstruct gene networks, cognitive systems for automatic text mining of scientific publications and databases are often employed. One such AI-driven platform, ANDSystem, is designed for automatic knowledge extraction of molecular interactions and, on this basis, the reconstruction of associative gene networks. The ANDSystem knowledge base contains information on more than 100 million interactions among diverse molecular genetic entities (genes, proteins, metabolites, drugs, etc.). The interactions span a wide range of types: regulatory relationships, physical interactions (protein–protein, protein–ligand), catalytic and chemical reactions, and associations among genes, phenotypes, diseases, and more. In the present study, we applied attention-based graph neural networks trained on the ANDSystem knowledge graph to predict new edges between proteins and ligands and to identify potential ligands for the SARS-CoV-2 ORF3a protein. The accessory protein ORF3a plays an important role in viral pathogenesis through ion-channel activity, induction of apoptosis, and the ability to modulate endolysosomal processes and the host innate immune response. Despite this broad functional spectrum, ORF3a has been explored far less as a pharmacological target than other viral proteins. Using a graph neural network, we predicted five small molecules of different origins (metabolites and a drug) that potentially interact with ORF3a: N-acetyl-D-glucosamine, 4-(benzoylamino)benzoic acid, austocystin D, bictegravirum, and L-threonine. Molecular docking and MM/GBSA affinity estimation indicate the potential ability of these compounds to form complexes with ORF3a. Localization analysis showed that the binding sites of bictegravir and 4-(benzoylamino)benzoic acid lie in a cytosolic surface pocket of the protein that is solvent-exposed; L-threonine binds within the intersubunit cleft of the dimer; and austocystin D and N-acetyl-D-glucosamine are positioned at the boundary between the cytosolic surface and the transmembrane region. The accessibility of these binding sites may be reduced by the influence of the lipid bilayer. The binding energetics for bictegravirum were more favorable than for 4-(benzoylamino)benzoic acid (docking score −7.37 kcal/mol; MM/GBSA ΔG −14.71 ± 3.12 kcal/mol), making bictegravirum a promising candidate for repurposing as an ORF3a inhibitor.

## Abstract (ru)

В последние годы методы искусственного интеллекта, основанные на анализе гетерогенных графов биомедицинских сетей, получили широкое распространение для предсказания молекулярных взаимодействий. В частности, графовые нейронные сети (graph neural networks, GNN) позволяют эффективно выявлять отсутствующие ребра в генных сетях, таких как сети белок-белковых взаимодействий, ген–заболевание, лекарство–мишень и др., и тем самым предсказывать новые биологические связи. Для реконструкции генных сетей часто применяют когнитивные системы автоматического анализа текстов научных публикаций и баз данных. Одна из таких платформ, базирующаяся на методах искусственного интеллекта,– ANDSystem, предназначенная для автоматического извлечения знаний о молекулярных взаимодействиях и на этой основе – реконструкции ассоциативных генных сетей. База знаний ANDSystem содержит сведения о более чем 100 млн взаимодействий между различными молекулярно-генетическими объектами (гены, белки, метаболиты, лекарства и др.). Взаимодействия представлены широким спектром типов: регуляторные связи, физические взаимодействия (белок–белок, белок–лиганд), каталитические и химические реакции, ассоциации между генами, фенотипами, заболеваниями и др. В настоящем исследовании мы применили графовые нейронные сети с механизмом внимания, обученные на графе знаний ANDSystem, для предсказания новых ребер между белками и лигандами и поиска потенциальных лигандов для белка ORF3a SARS-CoV-2. Вспомогательный белок ORF3a SARS-CoV-2 играет важную роль в патогенезе вируса за счет ион-канальной активности, индукции апоптоза и способности модулировать эндолизосомальные процессы и врожденный иммунитет хозяина. Несмотря на широкий спектр функций, ORF3a как фармакологическая мишень изучен значительно меньше, чем другие вирусные белки. Применение графовой нейронной сети позволило нам предсказать пять малых молекул разного происхождения (метаболиты и лекарство), потенциально взаимодействующих с ORF3a: N-ацетил-D-глюкозамин, 4-(бензоиламино)бензойная кислота, аустоцистин D, биктегравир и L-треонин. Молекулярный докинг и оценка аффинности методом MM/GBSA подтвердили потенциальную способность этих соединений образовывать комплексы с ORF3a. Анализ локализации показал, что сайты связывания биктегравира и 4-(бензоиламино)бензойной кислоты расположены в цитозольной поверхностной области белка, доступной растворителю; L-треонин связывается в межсубъединичной щели димера, а аустоцистин D и N-ацетил-D-глюкозамин – на границе между цитозольной поверхностью и трансмембранной областью. Доступность этих сайтов связывания может быть снижена из-за влияния липидного бислоя. Энергетические характеристики связывания у биктегравира по сравнению с 4-(бензоиламино)бензойной кислотой оказались более высокими (–7.37 ккал/моль в докинге; –14.71 ± 3.12 ккал/моль по MM/GBSA), что делает его перспективным кандидатом для репозиционирования как ингибитора ORF3a. Взаимодействие биктегравира с ORF3a может нарушать связывание ORF3а с белком хозяина VPS39 – субъединицей комплекса HOPS, участвующего в слиянии аутофагосом и поздних эндосом с лизосомами. Это, в свою очередь, может снимать индуцируемую ORF3a блокаду данного процесса и тем самым способствовать восстановлению аутофагического потока и лизосомной деградации вирусных компонентов.

## Introduction

The development of antiviral drugs is a priority due to the risk of global pandemics and the emergence of new variants of pathogenic viruses during such events, as demonstrated by the COVID-19 pandemic caused by SARS-CoV-2 (Ng et al., 2022). SARS-CoV-2 is an enveloped betacoronavirus with a positive-sense single-stranded RNA genome of approximately 29.9 kb; the genome encodes structural (S, E, M, N) as well as several nonstructural proteins that ensure replication and virion assembly (Naqvi et al., 2020). Because these proteins determine key stages of the viral life cycle, drug development efforts have focused primarily on three main targets: the main protease (3CLpro/Mpro), the RNA-dependent RNA polymerase (RdRp), and the S glycoprotein (Spike protein) (Boby et al., 2023).

A combination of experimental and computational approaches has been used to discover and optimize inhibitors of these targets: de novo design, high-throughput screening, and repurposing of known drugs (von Delft et al., 2023). This approach has yielded compounds with confirmed antiviral activity in vitro and in vivo and has enabled clinical strategies for treating COVID-19, including protease and polymerase inhibition. In particular, the antiviral nirmatrelvir/ritonavir (Paxlovid), which targets the main protease Nsp5 (nonstructural protein 5) of SARS-CoV-2, received full FDA approval on May 25, 2023, for the treatment of adults with COVID-19 (FDA, 2023). The drug remdesivir (Veklury), which targets the viral RNA-dependent RNA polymerase (RdRp, nsp12), was approved by the FDA in October 2020 (FDA, 2020). In parallel, alternative approaches are being developed to block fusion of the viral and cellular membranes during SARSCoV- 2 entry. In particular, peptide inhibitors complementary to the HR1/HR2 domains of the S2 subunit of the Spike protein prevent formation of the six-helix bundle (6-HB) – a key structure that mediates membrane fusion – and thereby block viral entry (Dong et al., 2024).

Among the promising classes of pharmacological targets are accessory viral proteins that modulate the interactions of SARS-CoV-2 with host cellular systems. One such protein is ORF3a. It is predominantly localized to late endosomes and lysosomes, where it co-localizes with the human lysosomal proteins LAMP1 and cathepsin D (Zhang J. et al., 2021; Hinkle et al., 2025). ORF3a forms ion channels (viroporin activity) (Zhang J. et al., 2022), induces apoptosis through oxidative stress and caspase activation (Zhang Y. et al., 2021), activates the NLRP3 inflammasome (the ORF3a–NLRP3–ASC cascade) (Zhang J. et al., 2022), and suppresses interferon signaling pathways, thereby enhancing viral pathogenicity (Zhang J. et al., 2022).

ORF3a is a dimeric membrane protein with three transmembrane helices and a large cytosolic C-terminal domain, as shown by cryo-EM (Kern et al., 2021). It interacts with the human protein VPS39 – a component of the HOPS complex – and this interaction blocks fusion of autophagosomes with lysosomes. A short tyrosine-based sorting signal motif, YXXΦ (Y, tyrosine; X, any amino acid; Φ, a hydrophobic residue), present in ORF3a as the sequence YNSV (residues 160–163), plays a key role in binding ORF3a to VPS39 (Stephens et al., 2025). The point mutation Y160A, which disrupts this motif, abolishes co-immunoprecipitation with VPS39 and lifts the block on autophagosome-lysosome fusion (Zhang Y. et al., 2021).

In recent years, artificial intelligence methods capable of uncovering hidden patterns in large biomedical datasets have seen increasingly widespread use in pharmacology and related fields. Graph neural networks (GNNs) are regarded as a particularly promising direction, as they enable the integration of heterogeneous biological information and the prediction of novel interactions in complex networks that have not previously been reported in the literature. An early study that played a notable role in shaping this approach was conducted by M. Zitnik et al. (2018), which showed that graph convolutional neural networks can model drug–disease interactions and predict drug side effects.

This approach has since advanced rapidly: studies have integrated diverse data sources (external databases, abstracts and full texts of scientific publications, patents, electronic medical records, etc.), predicted protein-ligand and protein–protein interactions, and identified targets for drug repurposing using GNNs (Stokes et al., 2020; Gaudelet et al., 2021). In particular, the compound halicin was identified as a candidate with antibacterial activity against resistant strains; using a graph neural network, this molecule was shown to have bactericidal effects against Mycobacterium tuberculosis, carbapenem-resistant Enterobacteriaceae, as well as multidrug-resistant strains of Acinetobacter baumannii, Pseudomonas aeruginosa, and Clostridioides difficile (Stokes et al., 2020).

Methods for reconstructing and analyzing gene and associative networks are increasingly used to identify pharmacological targets at the human genome scale (Ali, Alrashid, 2025). Against this backdrop, cognitive systems and knowledgeengineering methods that automate the extraction of facts from the literature and specialized databases – and construct biomedical knowledge graphs – are being actively developed. In such graphs, nodes represent genes, proteins, metabolites, diseases, drugs, and other biomedical entities, while edges represent their interactions (regulatory relationships, protein– protein interactions, disease associations, etc.). Notable resources implementing this approach include STRING (Nicholson, Greene, 2020; Szklarczyk et al., 2023), QIAGEN Ingenuity Pathway Analysis (Krämer et al., 2014), GeneGo/ MetaCore (Clarivate), and others

We previously developed the cognitive platform ANDSystem, designed for the reconstruction of associative gene networks. It brings together two strands: 1) automatic knowledge extraction from scientific publications and biological databases using semantic-linguistic templates and rules (Ivanisenko V.A. et al., 2015, 2019), and 2) integration of statistical and machine-learning methods, including graph neural networks, to predict and add new protein–protein interactions to the network (Ivanisenko N.V. et al., 2024).

The ANDSystem knowledge base (KB) contains information on more than 100 million interactions among various types of molecular genetic entities (genes, RNAs, proteins, metabolites, drugs), as well as cellular- and organism-level entities such as cells, biological processes, diseases, and phenotypic traits. Interactions are classified into 49 types, including regulatory relationships (regulation of expression, activity, stability, transport, etc.), physical interactions (protein– protein, protein–ligand), chemical interactions (catalytic reactions, post-translational modifications, etc.), and associative links (gene–disease, gene–phenotype, biological process– disease, etc.). Of particular note are “marker” relationships, which indicate that a gene, biological process, or phenotypic trait serves as an indicator of an associated disease or phenotype. In addition, the KB includes “risk factor” interactions, in which a gene, process, disease, phenotypic trait, or other entity is considered a risk factor for the associated disease (Ivanisenko V.A. et al., 2019).

A distinctive feature of ANDSystem is its web-based module ANDDigest, designed for searching and analyzing PubMed publications using ontological dictionaries (Ivanisenko T.V. et al., 2020, 2022). The module supports complex queries that simultaneously take into account multiple types of entities from the ANDSystem dictionaries, as well as userspecified refining keywords. Search results are presented in graphical form with in-text annotation of the detected entities, options for sorting and filtering (by date, source citation counts, and other parameters), visualization of the year-byyear dynamics of mentions of the annotated entities, and links to external databases

ANDSystem has been used to address a wide range of tasks based on the reconstruction and analysis of gene networks: reconstruction of the hepatitis C virus interactome (Saik et al., 2016); prioritization of genes associated with susceptibility to tuberculosis (Bragina et al., 2016); systems studies of preeclampsia (Glotov et al., 2015); analysis of the comorbidity of asthma and tuberculosis (Bragina et al., 2014); investigation of endothelial apoptosis in lymphedema (Saik et al., 2019); analysis of gene expression and the proteomic profile of clinical Helicobacter pylori strains associated with early stages of gastric cancer (Momynaliev et al., 2010); proteome stability in the Mars-500 project (Larina et al., 2015); interpretation of metabolomic data in studies of postoperative delirium (Ivanisenko V.A. et al., 2024); and the melanoma response to THz radiation (Butikova et al., 2025). Applying ANDSystem to the analysis of plasma metabolomic data from patients with COVID-19 made it possible to reconstruct gene networks describing the molecular genetic pathways through which SARS-CoV-2 proteins influence metabolic disturbances during infection (Ivanisenko V.A. et al., 2022). It was shown that nonstructural coronavirus proteins play a particularly important role in such networks.

In the present study, we used graph neural networks with an attention mechanism (Veličković et al., 2017) to predict new ligands of the ORF3a protein among metabolites and drugs represented in the ANDSystem knowledge base. Using a model we trained on the ANDSystem knowledge graph, five small molecules of endogenous and exogenous origin were predicted to potentially interact with ORF3a:

1. N-acetyl-D-glucosamine – a monomer of the natural polysaccharide chitin. According to molecular modeling data, it can form stable complexes with four SARS-CoV-2 proteins: the Spike protein (PDB ID: 6M0J), the nucleocapsid phosphoprotein N (PDB ID: 6WKP), the S protein (PDB ID: 6X79), and the 3CLpro protease (PDB ID: 7JVZ), and may potentially elicit an immune response against the virus (Baysal et al., 2021; Tekin, 2023). 2. 4-(benzoylamino)benzoic acid – an amide derivative of benzoic acid. This compound exhibits antiviral activity against and Rift Valley fever virus (Islam et al., 2018). 3. Austocystin D – a polyketide metabolite of fungi of the genus Aspergillus with cytotoxic and antineoplastic activity (Marks et al., 2011). 4. Bictegravir – a small-molecule integrase inhibitor used to treat HIV infection (Sax et al., 2023). Studies have shown its high binding affinity to the Spike protein (Ahsan, Sajib, 2021; Sun et al., 2021) and to the main protease of SARSCoV- 2 (Mpro, PDB ID: 6LU7) (Oner et al., 2023). 5. L-threonine – an essential amino acid involved in protein synthesis, glycosylation, and regulation of the immune response. Evidence indicates that L-threonine levels change in various viral infections, including COVID-19, reflecting metabolic reprogramming in response to infection (Barberis et al., 2020). Several studies have shown that amino acid profiles, including threonine, can serve as biomarkers of COVID-19 severity and are involved in regulating inflammatory responses and mucosal barrier functions (Páez- Franco et al., 2021).

Molecular docking and binding free energy calculations indicated that bictegravir and 4-(benzoylamino)benzoic acid are the most promising candidates for experimental validation. For bictegravir, binding energies of −7.37 kcal/mol (AutoDock Vina) and −14.71 ± 3.12 kcal/mol (MM/GBSA) were obtained, indicating higher affinity compared with 4-(benzoylamino) benzoic acid (−5.68 kcal/mol and −11.01 ± 3.58 kcal/mol, respectively). Bictegravir is therefore of particular interest as a candidate for drug repurposing studies

## Materials and methods

The ANDSystem cognitive system. ANDSystem is a cognitive platform for the automated extraction of facts and knowledge from scientific publication texts and factual databases, their integration into a unified ontological model (a knowledge graph), and the reconstruction of associative gene networks (Ivanisenko V.A. et al., 2015, 2019). In the knowledge graph, vertices correspond to molecular genetic entities (genes, RNA transcripts, proteins, metabolites, drugs) as well as cellularand organism-level objects (cell types, biological processes, diseases, phenotypic traits). Edges represent relationships between entities, including regulatory relationships (effects on expression, activity, stability, transport, etc.), physical contacts (protein–protein, protein–ligand interactions), chemical relationships (catalytic reactions, post-translational modifications, etc.), and associative links (gene–disease, gene–phenotype, process–disease, etc.). In its current version, the ANDSystem knowledge graph contains more than 1.5 million nodes and over 100 million edges.

For recognition of biomedical entity names and extraction of context-dependent relationships, ANDSystem uses more than 20,000 semantic linguistic templates and rules; in addition, large language models are employed, which improves the recall and precision of automated analysis of textual sources. To predict new interactions – particularly protein– protein interactions – graph neural networks (GNNs) trained on the ANDSystem knowledge graph, which is built from the scientific literature and specialized databases, are used (Ivanisenko T.V. et al., 2024).

ANDSystem includes the ANDDigest module – a specialized web-based system for searching and analyzing PubMed publications grounded in the ANDSystem ontological model and using dictionaries covering 13 types of biomedical entities (Ivanisenko T.V. et al., 2020, 2022). The ANDDigest database contains indexed and annotated PubMed texts, as well as computed characteristics and statistical co-occurrence measures for biomedical entities, which are used in subsequent stages of analysis and knowledge extraction

Obtaining vector representations of nodes in the ANDSystem knowledge graph. To compute vector representations of nodes in the ANDSystem knowledge graph, we used a graph neural network with an attention mechanism (GAT) based on TransformerConv (the PyTorch Geometric package, version 2.5.3) (Fey, Lenssen, 2019). The network architecture comprised four hidden layers with 256 neurons each. Every node in the ANDSystem knowledge graph was described by a 13-dimensional binary vector in which a value of “1” indicated the object’s membership in one of the 13 dictionary types defined by the ANDSystem ontology. Each edge was encoded by a 50-dimensional vector: the first 49 components corresponded to different interaction types and took values of 0 or 1 depending on whether the given type of relationship was present between the node pair in the knowledge graph, and the last component contained a numerical estimate of their co-occurrence (the p-value). This measure reflects the statistical significance of the joint mention of the object pair in PubMed abstracts and was computed using the ANDDigest module. The final node vector representations produced by the neural network had a dimensionality of 256

The attention mechanism in each hidden layer comprised four independent heads that computed the contribution of neighboring nodes, that is, nodes connected to the node under consideration by edges in the ANDSystem graph. In doing so, it took into account both the features of the neighboring nodes themselves and the features of the edges linking them (relationship types and the p-value). The loss function was the logistic loss (Mao et al., 2023) with a temperature parameter τ = 0.2. Parameters were optimized using AdamW (Zhou et al., 2024).

Given the large size of the ANDSystem knowledge graph, to speed up training, the model was not trained on the entire graph at once but on subgraphs automatically generated from it. For each target node, a subgraph was constructed that included the node itself and its neighbors within at most three hops. At each “neighborhood level” (i. e., at distances of 1, 2, or 3 hops), the number of neighboring nodes considered was limited: up to 15 at the first level, 10 at the second, and 5 at the third. These neighbors were selected at random.

The computations were performed on a workstation with six NVIDIA GeForce RTX 4090 GPUs (24 GB of memory each); all programs were written in Python version 3.12.11.

Fully connected neural network. To predict new interactions (edges) between proteins and metabolites in the ANDSystem knowledge graph, a fully connected neural network (multilayer perceptron) was used. The size of the input layer matched the dimensionality of the vector representation of a pair of nodes (512). The model architecture included three consecutive hidden layers with 512, 256, and 128 neurons. Each hidden layer used the Rectified Linear Unit activation function (ReLU) (Glorot et al., 2011):

**Function. 1.**
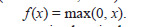
Function 1

The output layer contained a single neuron, the value of which reflected the probability of an edge existing between two nodes. For each protein–metabolite node pair, the neural network returned a value from 0 to 1, interpreted as the probability of an interaction between that pair. A standard threshold of 0.5 was used for classification: values above this threshold were interpreted as the presence of an interaction, and values below, as its absence (Harris, 2021).

From the ANDSystem knowledge graph, 250,000 object pairs were randomly selected, each consisting of one entity of type “protein” and the other of type “metabolite”; these pairs were treated as positive examples. As negative examples, an equal number of protein-metabolite pairs were randomly assembled from the set of all proteins and metabolites under the condition that the corresponding edge was absent from the original knowledge graph

For each pair (u, v), we constructed a composite feature vector of length 512 (with node embedding dimensionality d = 256), comprising four blocks: 1) vector representation of the protein eu; 2) vector representation of the metabolite ev; 3) element-wise absolute difference |eu – ev|; 4) element-wise product (Hadamard product) eu × ev.

The resulting array of vectors was split in an 80, 10, 10 % ratio into training, validation, and test subsets, respectively. The training subset was used to fit the model parameters during training; the test subset served for interim performance assessment and selection of the model’s optimal hyperparameters; and the validation subset was used only to evaluate the accuracy of the final model after training. In each subset, the ratio of positive to negative examples was 1:1.

The model’s performance after each training epoch (i. e., after the model had processed the entire training set) was evaluated on the test dataset using the Matthews correlation coefficient (MCC) (Chicco, Jurman, 2020), given by the formula:

**Formula. 1.**
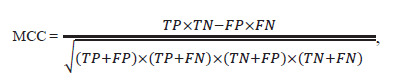
Formula1

where TP (true positives) – the number of object pairs correctly classified by the model as interacting; TN (true negatives) – the number of object pairs correctly classified by the model as non-interacting; FP (false positive) – the number of object pairs incorrectly classified by the model as interacting; FN (false negative) – the number of object pairs incorrectly classified by the model as non-interacting

Training was conducted over 83 epochs; the achieved MCC was 0.9542, indicating high model accuracy. The neural network was implemented using PyTorch version 2.4.1

Molecular docking was used for an initial assessment of affinity via the docking score (Vina score) and for building protein–ligand complex models. The Vina score used at this stage is an empirical estimate of the binding energy (kcal/mol); more negative values correspond to higher predicted affinity. Calculations were performed with AutoDock Vina 1.2.0 (Python API) (Trott, Olson, 2010; Eberhardt et al., 2021). Docking was carried out in a blind-docking mode, defining a search region that encompassed the entire surface of the ORF3a protein.

The most energetically favorable protein–ligand conformations (minimum Vina scores) were used as the starting structures for estimating the binding free energy (ΔG) by the MM/GBSA method.

MM/GBSA evaluation. ΔG was calculated using the AmberTools package (Case et al., 2023). The method accounts for molecular mechanics energies and solvation contributions (the generalized Born model) with a nonpolar component proportional to the solvent-accessible surface area, and provides an approximate thermodynamic descriptor of complex stability. The three-dimensional structure of the SARS-CoV-2 ORF3a protein was obtained from the Protein Data Bank (PDB ID: 6XDC).

## Results

**Prediction of new protein–ligand interactions using graph neural networks**

**Fig. 1.**
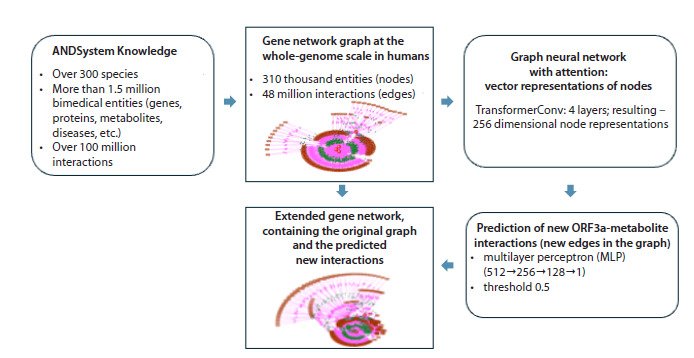
Schematic representation of the computational pipeline for predicting new interactions between human proteins and metabolites based on analysis of the ANDSystem knowledge graph.

The analysis workflow employed in ANDSystem to predict new interactions with graph neural networks is shown in Figure 1.

An associative human gene network at the whole-genome scale was exported from the ANDSystem knowledge base. The network included all 13 object types (including genes, proteins, metabolites, diseases, and others) and 49 interaction types (regulatory relationships: regulation of expression, activity, stability, transport, etc.; physical interactions: protein– protein, protein–ligand, etc.). In total, the graph contained about 310,000 nodes connected by 48 million edges. To obtain vector representations of nodes in the knowledge graph, a graph neural network with an attention mechanism was trained; an F1 score of 0.8003 was reached by epoch 230.

Based on the obtained vector representations of proteins and metabolites in the ANDSystem knowledge graph, a multilayer perceptron was trained as a binary classifier to predict edges missing from the graph. Training lasted 83 epochs; the achieved MCC was 0.9542. The trained model was then used to predict protein–metabolite edges for the ORF3a protein. In total, 38,172 potential links of this protein with small molecules of endogenous and exogenous origin were analyzed – including human metabolites and those of other organisms, as well as drugs, inorganic molecules, and ions – and five novel interactions not present in the ANDSystem knowledge base were identified.

**Fig. 2.**
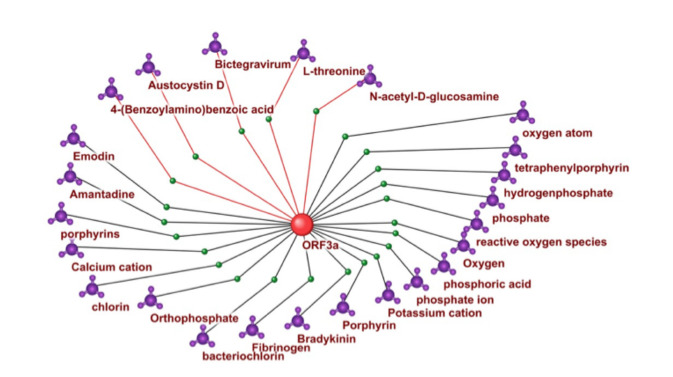
Interaction network of ORF3a with small molecules reconstructed using ANDSystem. Dark lines indicate interactions supported by scientific publications; red lines indicate interactions predicted by the graph neural network: N-acetyl-D-glucosamine, 4-(benzoylamino)benzoic acid, austocystin D, bictegravir, and L-threonine.

In Figure 2, the ORF3a interaction network is shown: edges initially present in the ANDSystem knowledge base are depicted in black, and new links predicted by the graph neural network and the binary classification model are shown in red. The knowledge base contained 19 interactions extracted from scientific publications, including both direct physical contacts and associative links between ORF3a and small molecules. For example, physical interactions experimentally confirmed by fluorescence and UV-visible spectroscopy were reported for chlorin and cationic porphyrins; in the same study, molecular docking indicated complex formation for related porphyrins (bacteriochlorin, tetraphenylporphyrin, TPP) (Lebedeva et al., 2021). As an example of an associative link, one can cite the ORF3a–bradykinin association discussed in the context of an intensified “bradykinin storm” via ORF3a/NS7b interaction in COVID-19 (Messina et al., 2021).

The group of predicted interactions comprised five candidates: N-acetyl-D-glucosamine (a chitin monomer and a precursor for glycosylation); 4-(benzoylamino)benzoic acid (a derivative of benzoic acid); austocystin D (a polyketide metabolite of Aspergillus fungi); bictegravir (an HIV integrase inhibitor; a medicinal drug); and L-threonine (an essential amino acid).

**Molecular docking and binding energy evaluation**

To assess the ability of the five predicted small molecules to physically interact with ORF3a, we performed molecular docking using AutoDock Vina and, for the resulting 3D complex models, recalculated the binding free energy (ΔG) by the MM/GBSA method (Table 1). The docking score (Vina score), which provides an empirical estimate of affinity, was used for the relative ranking of ligands, whereas the MM/GBSA ΔG values were considered an approximate thermodynamic descriptor of complex stability.

**Table 1.**
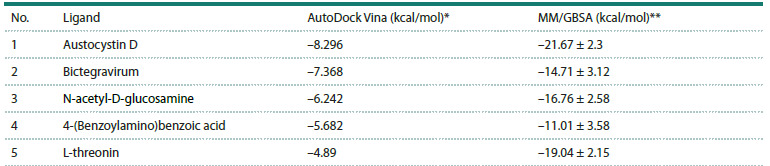
Calculated ORF3a–ligand binding metrics from AutoDock Vina and MM/GBSA * AutoDock Vina docking score (kcal/mol); ** binding free energy ΔG (kcal/mol) estimated by the MM/GBSA method.

According to AutoDock Vina, the highest predicted affinity was shown by austocystin D (−8.296 kcal/mol) and bictegravir (−7.368 kcal/mol); intermediate affinities, by N-acetyl-Dglucosamine (−6.242 kcal/mol) and 4-(benzoylamino)benzoic acid (−5.682 kcal/mol); and the lowest affinity, by L-threonine (−4.89 kcal/mol).

According to MM/GBSA, the most negative (i. e., lowest) ΔG was obtained for austocystin D (−21.67 ± 2.30 kcal/mol), followed by L-threonine (−19.04 ± 2.15) and N-acetyl-D-glucosamine (−16.76 ± 2.58), whereas bictegravir (−14.71 ± 3.12) and 4-(benzoylamino)benzoic acid (−11.01 ± 3.58) had ΔG values of smaller magnitude.

Taken together, the docking scores (Vina score) and the ΔG estimates from the MM/GBSA method indicate the potential formation of ORF3a complexes with the analyzed small molecules, serving as complementary criteria for the computational assessment of affinity.

**Fig. 3.**
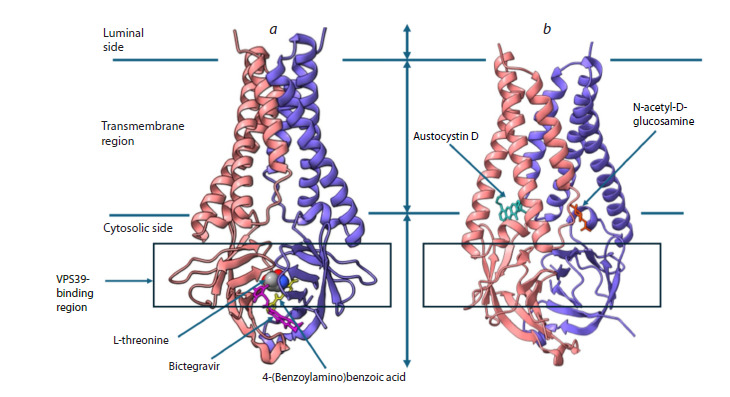
Spatial structures of ORF3a complexes with the analyzed ligands. a – ORF3a complex with L-threonine, bictegravir, and 4-(benzoylamino)benzoic acid; b – ORF3a complex with austocystin D and N-acetyl- D-glucosamine. The protein is shown in a ribbon representation; the two subunits of the dimer are colored differently. In panel (b), the protein structure is rotated to better display the ligands. Ligands are shown in a stick representation; their positions are indicated by arrows. L-threonine is shown in a space-filling (spheres) representation for clarity. Lines mark the regions of the protein corresponding to its position within the membrane (Kern et al., 2021): cytosolic side, transmembrane region, and luminal side (the lumen of the Golgi apparatus and endo-/lysosomes). The boxed area denotes the region involved in interaction with the VPS39 protein. Images were generated in ChimeraX.

The 3D models of ORF3a complexes with the ligands under study, constructed based on the results of molecular docking, are shown in Figure 3. According to cryo-EM data, ORF3a forms a dimer; each subunit contains three transmembrane helices and a large cytosolic C-terminal domain (Kern et al., 2021). ORF3a is predominantly localized to the membranes of the Golgi apparatus, endosomes, and lysosomes, participating in the regulation of vesicular transport and lysosomal exocytosis; it is also detected at the plasma membrane (Hinkle et al., 2025).

It is known that ORF3a interacts with VPS39 (the HOPS complex) and blocks the fusion of autophagosomes with lysosomes, leading to the accumulation of unfused autophagosomes and facilitating viral evasion of degradation (Zhang J. et al., 2021; Miller et al., 2023). For clarity, the corresponding region of the protein involved in the interaction with VPS39 is highlighted with a box in the Figure 3

**Fig. 4.**
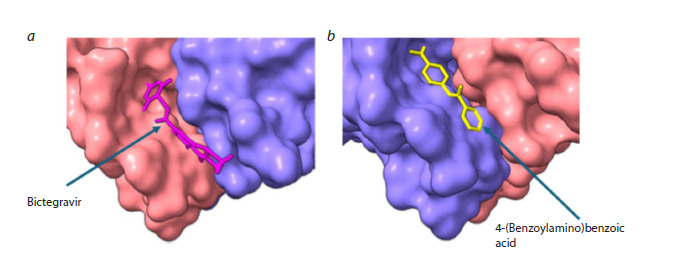
Surface of ORF3a bound to bictegravir (a) and 4-(benzoylamino)benzoic acid (b). Images were generated in ChimeraX.

According to the docking results, the binding sites of L-threonine, bictegravir, and 4-(benzoylamino)benzoic acid are located on the cytosolic surface of the dimer and partially overlap with the ORF3a–VPS39 binding region (Fig. 3a). L-threonine binds at the intersubunit interface (inter-subunit cleft) of ORF3a, is deeply buried there, and is essentially solvent-inaccessible. Bictegravir and 4-(benzoylamino) benzoic acid occupy solvent-exposed surface regions of the protein (Fig. 4). Austocystin D and N-acetyl-D-glucosamine bind at the boundary between the cytosolic surface and the transmembrane domain (Fig. 3b).

**Fig. 5.**
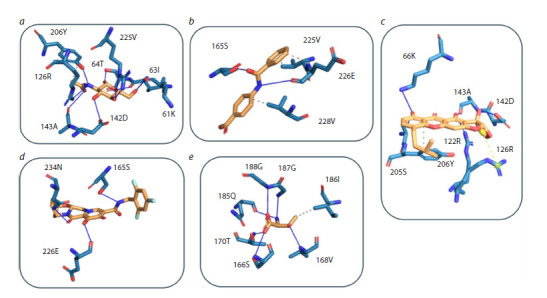
Detailed representation of the interactions of the analyzed ligands with ORF3a amino acid residues. a – N-acetyl-D-glucosamine; b – 4-(benzoylamino)benzoic acid; c –austocystin D; d – ictegravir; L-threonine. The ligand is shown in yellow and amino acid residues in blue. Hydrogen bonds are shown as solid lines; hydrophobic interactions are shown as dashed lines. Images were generated in PyMOL.

Details of hydrogen (H-) and hydrophobic contacts between the ligands and ORF3a amino acid residues are given in Table 2 and illustrated in Figure 5. N-acetyl-D-glucosamine forms multiple H-bonds with residues Lys61, Ile63, Thr64, Arg126, and others. 4-(Benzoylamino)benzoic acid forms H-bonds with Ser165 and Asp226, as well as hydrophobic contacts with Val225 and Val228. Austocystin D forms Hbonds with Ser165, Glu226, His227, and Asn234 and hydrophobic contacts with His227. Bictegravir forms three H-bonds (Ser165, Glu226, Asn234). L-threonine, located deep in the intersubunit cleft at the dimer interface, forms multiple H-bonds (with six residues) and hydrophobic contacts with Ile186.

**Table 2.**
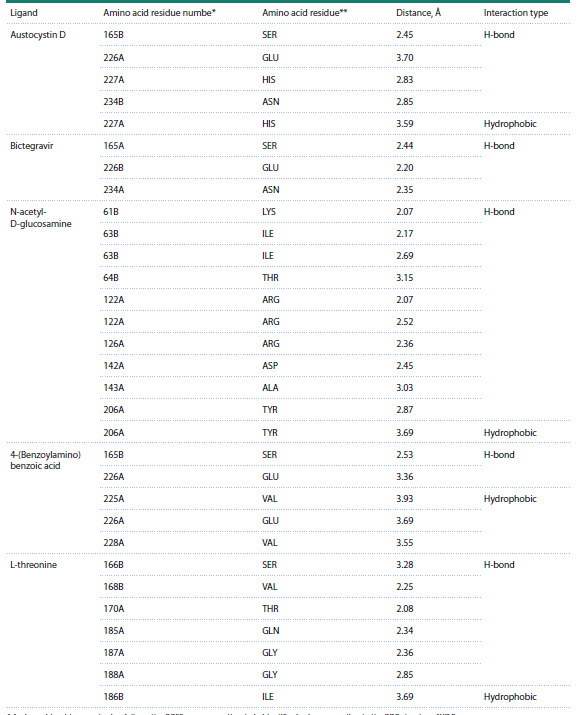
Molecular interactions of the ORF3a protein with ligands, obtained from analysis of the reconstructed ORF3a–ligand complexes using the PLIP (Protein-Ligand Interaction Profiler) web server * Amino acid residue numbering follows the ORF3a sequence; the chain identifier is given according to the PDB structure 6XDC. ** The amino acid involved in the interaction is indicated

## Discussion

Building on our previous work with GraphSAGE for predicting protein–protein interactions (Ivanisenko T.V. et al., 2024), in this study, we applied a graph neural network with an attention mechanism to predict interactions of the SARS-CoV-2 ORF3a protein with small molecules on the ANDSystem knowledge graph and identified five candidate ligands: N-acetyl-D-glucosamine, 4-(benzoylamino)benzoic acid, austocystin D, bictegravir, and L-threonine.

Unlike the GraphSAGE architecture, attention-based models update node representations by explicitly weighting the contributions of their neighbors: more informative relations receive higher weights, and less informative ones, lower weights. Multiple attention heads operate in parallel, and their outputs are then aggregated into the final node vector, enabling a more precise accounting of the local graph context (Wu et al., 2021).

To validate these predictions, we performed molecular docking and estimated the binding free energy (ΔG) of the protein–ligand complexes using the MM/GBSA method. The calculations showed that the predicted binding sites of austocystin D and N-acetyl-D-glucosamine are located at the boundary between the cytosolic surface and the transmembrane domain of ORF3a, whereas L-threonine, bictegravir, and 4-(benzoylamino)benzoic acid bind on the cytosolic side of the dimer; moreover, the binding regions of bictegravir and 4-(benzoylamino)benzoic acid partially overlap with the ORF3a–VPS39 interaction region.

The interaction of ORF3a with the host protein VPS39, a subunit of the homotypic fusion and protein sorting (HOPS) complex that regulates the late stages of endosome–lysosome compartment fusion, is well characterized (Zhang J. et al., 2021; Miller et al., 2023). It hinders the fusion of autophagosomes and late endosomes with lysosomes, thereby suppressing autophagic flux – a key pathway for the degradation of viral components.

The functional significance of the interaction interface between ORF3a and VPS39 is supported by the presence of an YXXΦ motif in the cytosolic domain of ORF3a (Y, tyrosine; X, any amino acid; Φ, a hydrophobic residue).

In ORF3a, this motif is present as the sequence YNSV (residues 160–163). Studies (Zhang J. et al., 2021; Miller et al., 2023) have shown that the point mutation Y160A disrupts co-immunoprecipitation of ORF3a with VPS39 and lifts the blockade of HOPS-dependent fusion, partially restoring autophagic flux.

It can be hypothesized that the predicted locations of the binding sites for bictegravir and 4-(benzoylamino)benzoic acid could influence the formation and/or stability of the ORF3a–VPS39 complex, making them promising candidates for functional intervention at the HOPS-dependent stage of autophagosome–lysosome fusion.

Taken together across metrics (Vina score and MM/ GBSA ΔG), bictegravir shows more negative values – indicating higher predicted affinity – than 4-(benzoylamino) benzoic acid (Vina score −7.37 kcal/mol and MM/GBSA ΔG −14.71 ± 3.12 kcal/mol vs. −5.68 kcal/mol and −11.01 ± 3.58 kcal/mol, respectively). In addition, bictegravir is a licensed HIV integrase inhibitor (the drug Biktarvy) (Gallant et al., 2017), making it a promising repurposing candidate. A potential mechanism of action for bictegravir as a therapeutic for COVID-19 could be inhibition of the ORF3a interaction with the host protein VPS39, which in turn would neutralize ORF3a’s ability to block fusion of endosome– lysosome compartments and promote degradation of viral components in lysosomes. In turn, 4-(benzoylamino)benzoic acid may be of interest as an aromatic carboxamide fragment for targeting protein–protein interaction interfaces within the ORF3a structure (Marks et al., 2011).

## Conclusion

Our approach – predicting new protein–ligand interactions on the ANDSystem knowledge graph followed by molecular docking and estimation of binding ΔG via the MM/GBSA method – enabled us to identify promising small-molecule ligand candidates for the SARS-CoV-2 ORF3a protein. Among the selected compounds, bictegravir and 4-(benzoylamino) benzoic acid are of greatest interest: their predicted sites lie on the cytosolic surface of ORF3a and partially overlap with the ORF3a–VPS39 interaction region. Based on energetic estimates, bictegravir shows more negative Vina score and ΔG values: AutoDock Vina, −7.37 kcal/mol; MM/GBSA, −14.71 ± 3.12 kcal/mol. For 4-(benzoylamino) benzoic acid, comparable but smaller-magnitude values were obtained: −5.68 kcal/mol and −11.01 ± 3.58 kcal/mol, respectively.

A limitation of this study is the lack of explicit consideration of the lipid bilayer: the calculations were performed without embedding the protein in a membrane, which may affect the conformation of ORF3a and the energetic contributions associated with ligand penetration into the hydrophobic environment. As a next step, molecular dynamics in a membrane model with recalculation of binding energies could be performed, followed by experimental validation of the results.

Taken together, the in silico results identify bictegravir as a priority candidate for experimental studies of its interaction with ORF3a – including within a drug-repurposing framework – and provide a foundation for further optimization of small molecules targeting this protein.

## Conflict of interest

The authors declare no conflict of interest.
